# Efficacy of WuSheZhiYang Pills in Mild-to-Moderate Atopic Dermatitis: Protocol for a Double-Blind, Randomized, Placebo-Controlled Trial

**DOI:** 10.2196/77927

**Published:** 2025-07-02

**Authors:** Xiangjin Gao, Yuning Ding, Ruiqi Cai, Xiuqi Zhang, Zhen Duan, Quanruo Xu, Fanlingzi Shen, Siyuan Li, Rui Zhang, Ruiping Wang

**Affiliations:** 1 Clinical Research Center Shanghai Skin Disease Hospital Shanghai China; 2 School of Medicine Tongji University Shanghai China; 3 School of Public Health Shanghai University of Traditional Chinese Medicine Shanghai China

**Keywords:** atopic dermatitis, WSZY pills, randomized controlled trial, clinical trial protocol, skin diseases

## Abstract

**Background:**

Atopic dermatitis (AD) is a recurrent, inflammatory, and chronic skin disease that influences over 200 million individuals around the world and is viewed as an important health problem due to its elevated prevalence, long course of disease, and heavy disease burden. WuSheZhiYang (WSZY) pills are composed of 11 Chinese herbs and have the effects of nourishing the blood, drying dampness, and relieving itching. In clinical practice, WSZY pills are recommended for itching skin diseases, but high-quality clinical trial evidence is still limited.

**Objective:**

In this study, we will implement a double-blind, randomized, placebo-controlled trial to evaluate the efficacy of WSZY pills for mild-to-moderate AD. We hypothesized that WSZY pills can effectively alleviate the disease condition of patients with mild-to-moderate AD.

**Methods:**

In this study, we will recruit 60 patients with mild-to-moderate in Shanghai Skin Diseases Hospital from December 2024 through December 2025. In this study, 60 male and female patients with AD aged 18 years to 65 years will be randomly assigned (2:1) to the treatment group (urea ointment and WSZY pills; n=40) or control group (urea ointment and placebo WSZY pills; n=20), and both groups will receive 4 weeks of treatment and 4 weeks of follow-up. The treatment group will receive 3 sessions of urea ointment and 7.5 g of WSZY pills each day for 4 weeks, and the control group will also receive 3 sessions of urea ointment and 7.5 g of placebo WSZY pills each day for 4 weeks. The primary indicator is the change in the objective Scoring Atopic Dermatitis (SCORAD) score between baseline and week 4. The secondary indicators include SCORAD at week 2 and week 8; Peak Pruritus Numerical Rating Scale (PP-NRS), Investigator's Global Assessment (IGA), Patient-Oriented Eczema Measure (POEM), and Dermatology Life Quality Index (DLQI) at week 2, week 4, and week 8; and the proportion of participants receiving remedial treatment, amount of levocetirizine tablets used, and recurrence rate at week 8. In this study, we will analyze the full analysis set and per-protocol set using SAS software version 9.4, and a 2-tailed alpha level of .05 will be viewed as statistically significant.

**Results:**

The study received ethics permission in September 2024, and trial registration was completed in October 2024. Recruitment started in December 2024 and is expected to be completed by December 2025. As of June 2025, 30 participants with mild-to-moderate AD were enrolled. Data analysis will begin in January 2026. The main results of the trial are expected to be submitted for publication in peer-reviewed scientific journals in the summer of 2026.

**Conclusions:**

This study will evaluate the efficacy of WSZY pills for AD and provide additional evidence, suggest new therapeutic options for patients, and reduce their disease burden.

**Trial Registration:**

International Traditional Medicine Clinical Trial Registry ITMCTR2024000724; http://itmctr.ccebtcm.org.cn/zh-CN/Home/ProjectView?pid=bda070f8-a733-4f52-87b0-39e4be57ac00

**International Registered Report Identifier (IRRID):**

DERR1-10.2196/77927

## Introduction

### Background

Atopic dermatitis (AD), a chronic inflammatory skin disorder characterized by chronic eczematous lesions and intense pruritus, affects more than 200 million individuals worldwide [1]. Although the prevalence of AD in China has increased later than in western higher-income countries, it has increased rapidly in the past 10 years. Globally, AD is associated with a heavy disease burden, and it is the skin disease with the highest disease burden among nonfatal diseases [2]. AD has become an important health problem due to its elevated prevalence, long course of disease, and heavy disease burden.

AD can cause not only skin lesions, itch, pain, and sleep disorders but also a heavy psychological burden. Clinical practice often aims to alleviate clinical symptoms, reduce recurrence, reduce complications, and improve life satisfaction in patients [3]. Many treatment methods for ADs have been proposed, such as emollients containing urea, antihistamines, corticosteroids, antimicrobial agents, and immunotherapy [4-6]. Corticosteroids are the main treatment method, but their use is limited due to the susceptibility to cause adverse reactions [7-11].

Traditional Chinese medicine (TCM) has been systematically used in AD treatment and management and has shown good effects at alleviating the disease condition, reducing disease recurrence, improving quality of life, and causing fewer adverse reactions [12]. WuSheZhiYang (WSZY) pills, part of TCM, are composed of 11 herbs, including *Zaocys dhumnades*, *Saposhnikovia divaricata*, snake bile, cortex *Phellodendri amurensis*, rhizoma atractylodis, radix ginseng, fructus cnidii, *Sophora flavescens*, artificial bezoar, and *Angelica sinensis*. They are used orally and have the effects of nourishing the blood, dispelling wind, drying dampness, and relieving itching. Nourishing the blood means that it improves skin dryness and desquamation caused by blood deficiency; dispelling wind means that it evacuates pathogens, reduces skin irritation caused by pathogens, and alleviates pruritus; drying dampness means that it clears heat and eliminates dampness in the body to reduce eczema exudation; and relieving itching means that it directly inhibits itching symptoms caused by pathogens. WSZY pills are usually prescribed for patients with mild-to-moderate itching skin diseases (eczema, urticaria, pruritus, neurodermatitis, seborrheic dermatitis, pityriasis rosea, psoriasis vulgaris) in clinical practice but without high-quality clinical trial evidence for mild-to-moderate AD. Therefore, we will carry out exploratory research with patients with mild-to-moderate AD.

### Objectives

In this study, we plan to implement a double-blind, randomized, placebo-controlled clinical trial to evaluate the efficacy of WSZY pills for mild-to-moderate AD. Our primary objective is to evaluate the effect of WSZY pills on the severity of AD and recurrence rates in patients with mild-to-moderate AD. The secondary objectives are to assess the effect of WSZY pills on itching level, quality of life, and safety in patients with mild-to-moderate AD. This trial will provide additional evidence for the clinical efficacy of WSZY pills for AD treatment, which may provide a new treatment option for patients with mild-to-moderate AD. We hypothesized that WSZY pills can effectively alleviate the disease condition of patients with mild-to-moderate AD.

## Methods

### Study Design

**Figure 1:**
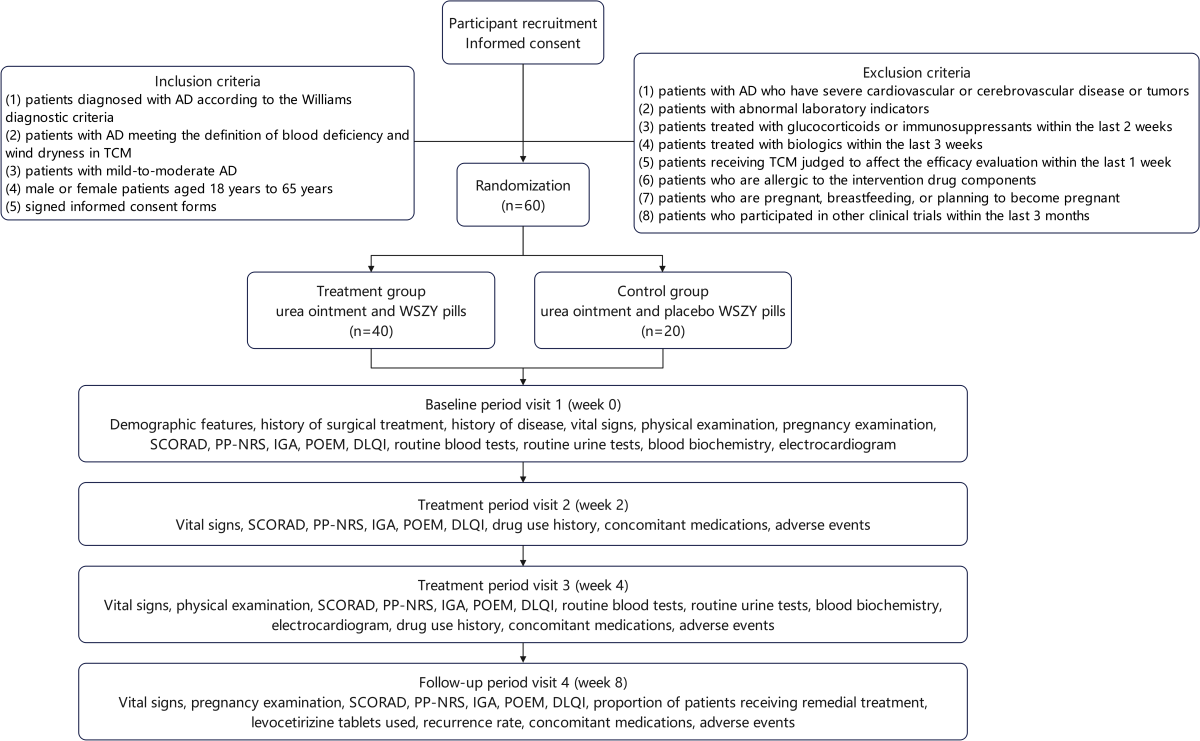
Clinical trial flowchart. AD: atopic dermatitis; DLQI: Dermatology Life Quality Index; IGA: Investigator's Global Assessment; POEM: Patient-Oriented Eczema Measure; PP-NRS: Peak Pruritus Numerical Rating Scale; SCORAD: Scoring Atopic Dermatitis; TCM: traditional Chinese medicine; WSZY: WuSheZhiYang.

This study is a double-blind, randomized, placebo-controlled trial to evaluate the efficacy of WSZY pills for AD treatment. It was designed in line with the SPIRIT (Standard Protocol Items: Recommendations for Interventional Trials) statement [13]. Patients with mild-to-moderate AD will be recruited in inpatient and outpatient departments in Shanghai Skin Disease Hospital from December 2024 through December 2025. In this study, 60 patients with mild-to-moderate AD will be randomly assigned (2:1) to the treatment group (urea ointment and WSZY pills; n=40) or control group (urea ointment and placebo WSZY pills; n=20). All participants in both groups will undergo a 4-week treatment phase followed by a 4-week observational follow-up to evaluate the treatment response and disease progression. The flowchart of this study is shown in Figure 1.

### Inclusion Criteria

The inclusion criteria in this study are as follows: (1) patients diagnosed with AD according to the Williams diagnostic criteria [14], (2) patients with AD meeting the definition of blood deficiency and wind dryness in TCM [15], (3) patients with mild-to-moderate AD (objective Scoring Atopic Dermatitis [SCORAD] score ≤50, body surface area ≤10%), (4) male or female patients aged 18 years to 65 years, and (5) signed informed consent forms.

The main Williams diagnostic criterion is skin itching, while the secondary criteria are (1) a history of dermatitis and eczema on the flexed side, including the elbow fossa, popliteal fossa, ankle anterior, and neck; (2) history of asthma or allergic rhinitis; (3) history of dry skin on the entire body in recent years; (4) eczema on the flexor side (eczema on the cheeks or forehead and extensor sides of the limbs in children younger than 4 years), and (5) onset before the age of 2 years (for patients older than 4 years) [14]. A confirmed diagnosis for AD involves meeting the main criterion plus 3 or more of the secondary criteria.

### Exclusion Criteria

In this study, the exclusion criteria include (1) patients with AD who have severe cardiovascular or cerebrovascular disease or tumors; (2) patients with abnormal laboratory indicators (serum creatinine, aspartate aminotransferase, alanine aminotransferase, gamma glutamyl transpeptidase, alkaline phosphatase, and total bilirubin); (3) patients treated with glucocorticoids or immunosuppressants within the last 2 weeks; (4) patients treated with biologics within the last 3 weeks; (5) patients receiving TCM judged to affect the efficacy evaluation within the last 1 week; (6) patients who are allergic to the intervention drug components; (7) patients who are pregnant, breastfeeding, or planning to become pregnant; and (8) patients who participated in other clinical trials within the last 3 months.

### Sample Size

In this study, the sample size was calculated based on the difference in the change in SCORAD score from baseline (week 0) to week 4, as the primary indicator, between the treatment group (urea ointment and WSZY pills) and control group (urea ointment and placebo WSZY pills). Based on previous evidence on the efficacy of TCM for AD treatment, we used a difference of 9.8 (SD 12) points [16]. ‌Sample size estimation‌ was performed using PASS 15.0 software (NCSS Statistical Software). Using the SD of 12.0, the sample size calculation for the 2:1 randomization design indicated that 36 patients with AD in the treatment group and 18 patients with AD in the control group would provide 80% statistical power to detect a difference of 9.8 points in the improvement in the SCORAD score at a 2-tailed α level of .05. Considering a dropout rate of 10%, we plan to enroll 40 patients with AD and 20 patients with AD in the treatment group and control group, respectively.

### Randomization

In this study, patients with AD will be randomly allocated at a 2:1 ratio to either the treatment group or the control group. We will use cluster randomization with combined blocks of 4 and 6 to ensure a balanced group allocation. An independent statistician will construct the computer-generated randomization sequences using SAS software (version 9.4; SAS Institute Inc). To ensure allocation concealment, the generated allocation schedules will be sealed in sequentially numbered, light-resistant envelopes and securely maintained by an independent trial coordinating center.

### Blinding

In this study, patients with AD, dermatologists (investigators), data collectors, and the statistician will be blinded to the treatment allocation during the entire trial period to prevent bias. Rigorous allocation concealment is preserved through physical separation of randomization records from study operations. This comprehensive blinding strategy aims to mitigate bias during delivery of the intervention, endpoint evaluation, and statistical interpretation.

### Interventions

In this study, patients with AD in the treatment group will receive 3 sessions of urea ointment and 7.5 g of WSZY pills each day for 4 weeks. To ensure the correct application of urea ointment, we will instruct the patients to apply urea ointment to the affected area and instruct patients that one knuckle length of urea ointment should be used to cover no more than 1% of the body surface area. This should be followed by a finger massage of the area for 3 minutes to 5 minutes to promote absorption. To ensure the correct administration of WSZY pills, we will instruct patients to take 1 bag (2.5 g) of the WSZY pills per dose, 3 times a day with water.

Patients with AD in the control group will also receive 3 sessions of urea ointment and 7.5 g of placebo WSZY pills each day for 4 weeks. The placebo WSZY pills are made of dextrin and are produced and provided by the same company, which maintains complete consistency with the WSZY pills in terms of appearance, weight, color, odor, and package. The application, individual guidance, and instruction for placebo WSZY pill administration among patients in the control group is completely in line with that in the treatment group.

In this study, patients will be instructed not to use any additional treatment for AD. If a patient uses additional treatment for an emergency condition, detailed information should be recorded, and the patient should be withdrawn.

### Outcomes

#### Primary Outcome

The primary outcome indicator in this study is the difference in the SCORAD score change from baseline (week 0) to week 4. The SCORAD index is made of the affected body surface area, skin lesion severity, and patient self-assessment of itching and quality of sleep. It is an evaluation method to assess the severity of AD as objectively as possible.

The SCORAD score includes the doctor's assessment (A, B) and the patient's own assessment (C), which is the most comprehensive evaluation method. (A) involves evaluating the affected area, including the head and neck area (accounting for 9%), both upper limbs (accounting for 9% each), both lower limbs (accounting for 18% each), the front and back of the trunk (accounting for 18% each), and the genital area (accounting for 1%). (B) involves 6 clinical features, including erythema, edema or papules, exudation or scabbing, epidermal peeling, lichenification, and dryness. Each feature is scored according to its severity, ranging from 0 to 3 points for each item. The patient self-assessment (C) includes itching and sleep deprivation in the last 3 days. The SCORAD score is calculated as A/5+7×B/2+C, with the total score ranging from 0 to 103 points. A higher score indicates a more severe disease condition. According to the score, the condition is considered mild (0-24 points), moderate (25-50 points), or severe (>50 points) [17]. In this study, the SCORAD will be estimated at baseline (week 0), week 2, week 4, and week 8.

#### Secondary Outcomes

##### Overview

The secondary outcome indicators include the SCORAD score at week 2 and week 8; Peak Pruritus Numerical Rating Scale (PP-NRS), Investigator’s Global Assessment (IGA), Patient-Oriented Eczema Measure (POEM), and Dermatology Life Quality Index (DLQI) at week 2, week 4, and week 8; and the proportion of participants receiving remedial treatment, the amount of levocetirizine tablets used, and the recurrence rate at week 8 [18,19]. A comprehensive study schedule is provided in Table 1, and details of secondary outcome indicators are included.

**Table 1:** Study schedule of the WuSheZhiYang (WSZY) pill clinical trial (8 weeks).

Indicator	Baseline	Treatment		Follow-up
	Visit 1 (week 0)	Visit 2 (week 2)	Visit 3 (week 4)	Visit 4 (week 8)
Informed consent	✓	—^a^	—	—
Demographic feature	✓	—	—	—
History of surgical treatment	✓	—	—	—
History of disease	✓	—	—	—
Vital sign	✓	✓	✓	✓
Physical examination	✓	—	✓	—
Pregnancy examination	✓	—	—	✓
Inclusion/exclusion criteria check	✓	—	—	—
Randomization	✓	—	—	—
SCORAD^b^	✓	✓	✓	✓
PP-NRS^c^	✓	✓	✓	✓
IGA^d^	✓	✓	✓	✓
POEM^e^	✓	✓	✓	✓
DLQI^f^	✓	✓	✓	✓
Proportion of participants receiving remedial treatment	—	—	—	✓
Levocetirizine tablets used	—	—	—	✓
Recurrence rate	—	—	—	✓
Routine blood tests	✓	—	✓	—
Route urine tests	✓	—	✓	—
Blood biochemistry	✓	—	✓	—
Electrocardiogram	✓	—	✓	—
Drug use history	—	✓	✓	—
Concomitant medications	—	✓	✓	✓
Adverse events	—	✓	✓	✓

^a^Not applicable.

^b^SCORAD: Scoring Atopic Dermatitis.

^c^PP-NRS: Peak Pruritus Numerical Rating Scale.

^d^IGA: Investigator’s Global Assessment.

^e^POEM: Patient-Oriented Eczema Measure.

^f^DLQI: Dermatology Life Quality Index.

##### PP-NRS

Pruritus severity in patients with mild-to-moderate AD will be quantified using the validated PP-NRS, which is an 11-point patient-reported instrument (0=no pruritus; 10=extreme pruritus). Participants will self-report their maximum itch intensity over the preceding 24 hours using this standardized metric. Scores of PP-NRS will be stratified as 0 (none), 1-3 (mild), 4-6 (moderate), 7-8 (severe), and 9-10 (extremely severe). In this study, the PP-NRS will be evaluated at baseline (week 0), week 2, week 4, and week 8.

##### IGA

The IGA is a rough score based on the doctor's subjective judgment and allows researchers to quickly evaluate the severity of disease. This validated 6-point ordinal scale assesses the following key clinical features: no erythema (0), minimal erythema with barely perceptible edema/papules (1), mild erythema with detectable edema/papules (2), moderate erythema with evident edema/papules (3), severe erythema with marked edema/papules (4), and extreme skin involvement with confluent erythema and edema (5). Longitudinal assessments of the IGA will be implemented at baseline (week 0), week 2, week 4, and week 8.

##### POEM

POEM focuses on the frequency and severity of 7 symptoms (itching, sleep disorders, bleeding, exudation, cracking, scales, and dryness) in the last week through the patient’s own assessment. All items are scored using a 5-point rating system: 0 (none), 1 (mild), 2 (moderate), 3 (severe), and 4 (extremely severe). The total POEM score (range: 0-28) is derived by cumulatively scoring the individual items, with a higher score indicating more severe illness. In this study, POEM will be evaluated at baseline (week 0), week 2, week 4, and week 8.

##### DLQI

The DLQI systematically evaluates impairment of quality of life in the last week across 6 key domains, including physiological response, psychological feeling, family, interpersonal communication, occupational restrictions, social activities, and treatment response [20]. Using a 4-point severity scale (0=none, 1=mild, 2=severe, 3=extremely severe) across 10 relevant items, the total score of the DLQI (range: 0-30) is calculated by summing the 10 items, with a higher DLQI score indicating a lower dermatology quality of life. In this study, the DLQI will be evaluated at baseline (week 0), week 2, week 4, and week 8.

### Data Collection and Management

Standardized data collection will be implemented using a case report form (CRF) completed by trained dermatologists. The CRF includes 6 parts: (1) demographic characteristics, covering age, height, and weight; (2) history of surgical treatment and diseases; (3) vital signs and physical examination, including blood pressure, pulse, height, and body weight; (4) severity indicators of atopic dermatitis (SCORAD, PP-NRS, IGA, POEM, DLQI) at baseline (week 0), week 2, week 4, and week 8; (5) pregnancy examination, routine blood tests, routine urine tests, blood biochemistry, and an electrocardiogram; and (6) drug use history, concomitant medications, and adverse events.

The collected data will be uniformly reviewed, coded, and sampled by professional personnel. The database will be established using Epidata 3.1 software, where input validation procedures (including variable type definitions, value ranges, and skip patterns) will be configured for each variable. All data will be entered using the double data entry method by two independent operators. Consistency checks will be performed on the two generated databases. For any inconsistent variables, the original CRF will be consulted to identify and correct errors in the database until complete consistency is reached between both entries. The finalized database will be converted to a SAS data set. Logical checks and validation will be performed using SAS 9.4 software.

### Statistical Analysis

All data will be systematically recorded in the CRF and subsequently entered into a dual-validated electronic database using EpiData software 3.1, with final analyses performed using SAS 9.4. Quantitative variables that are normally distributed will be described using mean and SD, and those a skewed distribution will be described using median and interquartile range. Student *t* tests or Wilcoxon rank-sum tests will be used to compare the differences between groups for quantitative variables as appropriate, depending on the data distribution. Categorical variables will be presented as frequencies and proportions (%), and chi-square tests will be used to compare the differences in categorical variables between the groups. In addition, when the expected frequency is low (<1), Fisher exact tests will be used to compare the differences in categorical variables between the groups. In this study, a 2-tailed α level of .05 will be viewed as statistically significant. Statistical analyses will follow the intention-to-treat principle, including all randomly assigned patients regardless of protocol adherence or study completion [21]. We will handle missing data based on the assumption of missing-at-random and use the sequential regression multiple imputation method, which imputes missing values using regression models for each variable, conditional on the other variables in the data [22]. Additionally, analyses will be performed on both the full analysis set and the per-protocol set.

### Safety Assessment

Prior to enrollment in this study, all patients diagnosed with mild-to-moderate AD will receive a comprehensive risk disclosure through a standardized informed consent process. Adverse events are defined as any adverse medical occurrences following administration of the treatment, encompassing symptoms, physical signs, diagnosable diseases, or laboratory abnormalities, regardless of causality assessment. Participants will be required to report all adverse events occurring during the 4-week treatment period and the subsequent 4-week follow-up. All reported events will be systematically documented in the CRF with precise temporal and descriptive details. In accordance with clinical practice guidelines, any participant experiencing a severe adverse event will undergo immediate study discontinuation and receive protocol-specified medical management at no cost to the participant. This safety protocol ensures both rigorous data collection and participant welfare throughout the trial duration. Given that this trial presents minimal risks, we will not establish a data monitoring committee.

### Ethical Considerations

This clinical trial protocol (version 1.0, 2024/08/08) was approved by the Institutional Review Board of Shanghai Skin Disease Hospital (approval number 2024-40) and was registered on the International Traditional Medicine Clinical Trials Registry. This study is conducted in strict compliance with the ethical principles outlined in the Declaration of Helsinki and its subsequent amendments. Written informed consent is obtained from all participants prior to their participation in the study. For any participants who are unable to provide informed consent, written consent is obtained from their legal guardian. Participants will be compensated for their time and effort during the study, with total compensation amounting to ¥800 (US $111.36) per participant.

All signed informed consent forms as well as all collected CRFs are stored in a locked place, accessible only by the researchers of this study. All data are stored in Shanghai Skin Disease Hospital for 10 years until the end of the study. After that, all data are to be destroyed.

## Results

The study received ethics permission in September 2024, and trial registration was completed in October 2024. Recruitment started in December 2024 and is expected to be completed by December 2025. As of June 2025, 30 participants with mild-to-moderate AD were enrolled. Data analysis will begin in January 2026. The main results of the trial are expected to be submitted for publication in peer-reviewed scientific journals in the summer of 2026.

## Discussion

### Summary

To our knowledge, this is the first clinical trial to use WSZY pills to treat AD. This study explores the efficacy of WSZY pills for AD treatment, with the goal of providing additional effective treatment recommendations. The expected results of this study are that the WSZY pills combined with urea ointment can effectively alleviate the disease condition of patients with mild-to-moderate AD.

AD is an itchy, inflammatory, and chronic skin dermatitis affecting both children and adults and is one of the most common chronic skin diseases [23,24]. AD usually exhibits a chronic course with thickening of skin, sleep disturbance, severe interference of quality of life, and impact on daily activities, which not only cause physical pain for patients but also impose heavy social and economic burdens [25]. Modern pharmacological studies indicate that the ingredients of WSZY pills have immunomodulatory and anti-inflammatory properties. For example, the water extracted from *Zaocys dhumnade* has immunomodulatory, anti-inflammatory, and capillary permeability–reducing effects. Oxymatrine in *Sophora flavescens* has immunosuppressive effects, and flavonoids and polysaccharides have anti-inflammatory effects [26,27]. Although the application of TCM has a long history in the treatment of AD in China, high-quality clinical trial evidence is still needed to demonstrate its efficacy and safety [2,28,29].

WSZY pills are part of TCM and have been used in clinical practice for more than 30 years. Previous studies have shown that the combination of WSZY pills and ebastine tablets can alleviate itching, can improve erythema and exudation, can reduce recurrence, and has a good safety profile [30]. In addition, the combination of WSZY pills and dehumidification Zhiyang ointment can improve acute eczema, alleviate the stimulating effect of inflammatory factors, reduce recurrence, and result in fewer adverse reactions [31]. Therefore, the risk associated with the intervention group receiving WSZY pills is limited. Moreover, basic treatment (urea ointment) and remedial treatment (if severe itching occurs, levocetirizine tablets will be administrated) are also included in the study design, which ensures the safety of all patients with AD enrolled in this study.

Findings in this study are expected to provide several key points for AD treatment. The research results cover the relationship between the administration of WSZY pills and changes in SCORAD, PP-NRS, IGA, POEM, and DLQI scores, as well as the recurrence rate in patients with AD. The aim is to explore the effects of WSZY pills on the severity of AD, itching level, quality of life, and recurrence rate in patients with AD, thereby evaluating their efficacy. Based on these findings, we can recommend an additional treatment option for patients with AD, which will help alleviate their condition, reduce their medical burden, and facilitate long-term treatment and management. In all, this clinical trial will provide additional evidence for the clinical efficacy of WSZY pills for AD treatment and provide a new treatment option for patients with mild-to-moderate AD, which can also alleviate their disease condition and reduce their medical burden. We will share our results through publications covering skin problems, as well as through high-impact, internationally peer-reviewed journals.

### Limitations

There are some limitations in this study. All patients with mild-to-moderate AD in this study will be recruited in Shanghai Skin Diseases Hospital, which results in high internal validity but may limit external generalizability. In addition, patients with AD recruited in this study must meet the definition of the syndrome of blood deficiency and wind dryness in TCM, which also may limit external generalizability of the research results to other patients with AD. In addition, all participants will undergo a 4-week treatment followed by a 4-week observational follow-up in this study, which might lead to a relatively higher rate of loss to follow-up. Therefore, more relevant multicenter, randomized, controlled clinical trials need to be conducted in future.

### Conclusions

This study will evaluate the efficacy of WSZY pills for AD and provide additional evidence, suggest new therapeutic options for patients, and reduce their disease burden. The findings will provide evidence-based recommendations for integrating this traditional medicine into comprehensive AD management strategies, potentially offering patients an effective adjuvant therapy to alleviate clinical symptoms, reduce the financial burden associated with chronic treatment, and support the long-term management of this recalcitrant dermatological condition.

## Abbreviations

AD: atopic dermatitis
CRF: case report form
DLQI: Dermatology Life Quality Index
IGA: Investigator's Global Assessment
POEM: Patient-Oriented Eczema Measure
PP-NRS: Peak Pruritus Numerical Rating Scale
SCORAD: Scoring Atopic Dermatitis
SPIRIT: Standard Protocol Items: Recommendations for Interventional Trials
TCM: traditional Chinese medicine
WSZY: WuSheZhiYang

## Data Availability

The data sets generated or analyzed during this study are available from the corresponding author on reasonable request.
